# Raman Spectra Shift of Few-Layer IV-VI 2D Materials

**DOI:** 10.1038/s41598-019-55577-x

**Published:** 2019-12-20

**Authors:** Minwoo Park, Jin Sik Choi, Li Yang, Hoonkyung Lee

**Affiliations:** 1 Department of Physics, Washington University in St. Louis, St. Louis, Missouri 63136 USA; 2 Department of Physics, Konkuk University, Seoul, 05029 Korea

**Keywords:** Magnetic properties and materials, Structural properties, Two-dimensional materials

## Abstract

Raman spectroscopy is the most commonly used method to investigate structures of materials. Recently, few-layered IV-VI 2D materials (SnS, SnSe, GeS, and GeSe) have been found and ignited significant interest in electronic and optical applications. However, unlike few-layer graphene, in which its interlayer structures such as the number of its layers are confirmed through measurement of the Raman scattering, few-layer IV-VI 2D materials have not yet been developed to the point of understanding their interlayer structure. Here we performed first-principles calculations on Raman spectroscopy for few-layer IV-VI 2D materials. In addition to achieving consistent results with measurements of bulk structures, we revealed significant red and blue shifts of characteristic Raman modes up to 100 cm^−1^ associated with the layer number. These shifts of lattice vibrational modes originate from the change of the bond lengths between the metal atoms and chalcogen atoms through the change of the interlayer interactions. Particularly, our study shows weak covalent bonding between interlayers, making the evolution of Raman signals according to the thickness different from other vdW materials. Our results suggest a new way for obtaining information of layer structure of few-layer IV-VI 2D materials through Raman spectroscopy.

## Introduction

Recently, van der Waals (vdW) materials such as graphene and transition metal dichalcogenides have attracted substantial attention from nanoscience and technology fields because they have exotic electronic properties and potential applications for energy storage and harvest. In addition, their properties have been shown to be dependent on the number of layers they have^1–6^. For example, for graphene, it has a Dirac cone in a single layer while it is gone more than one layer, leading to different physical phenomena such as massless and mass Fermions, respectively^7,8^. Bilayer graphene shows an exotic superconducting phase as the twisting angle between the layers^9^. By contrast, single-layer MoS_2_ is semiconducting with a direct bandgap of 1.57 eV, and the properties of few-layer MoS_2_ are significantly tuned to be indirect-bandgap semiconductors by the number of layers, because of interlayer interaction^10–12^. Although the technology for controlling the thickness of vdW materials has been developed by exfoliation and atomic layer deposition methods^13–18^, it is highly appreciated to conveniently verify the number of layers or thicknesses of vdW materials for device applications.

As one of the most fundamental tools to study structures, Raman spectroscopy can be employed to determine the thickness of few-layer vdW materials such as graphene^19^ and transition metal dichalcogenides^20^ because the lattice vibration modes are dependent on the number of layers of the vdW materials. For instance, for graphene, the number of layers can be determined based on the results of Raman spectroscopy: the change of the position of G peak or the position and shape of the 2D band^21^. In the case of MoS_2_, the positions of the E^1^_2g_ and A_1g_ peaks provide clues for the thickness^22–24^. For black phosphorus, the intensities of A_g_^1^ and B_2g_ peaks play the same roles^25^.

**Figure 1:**
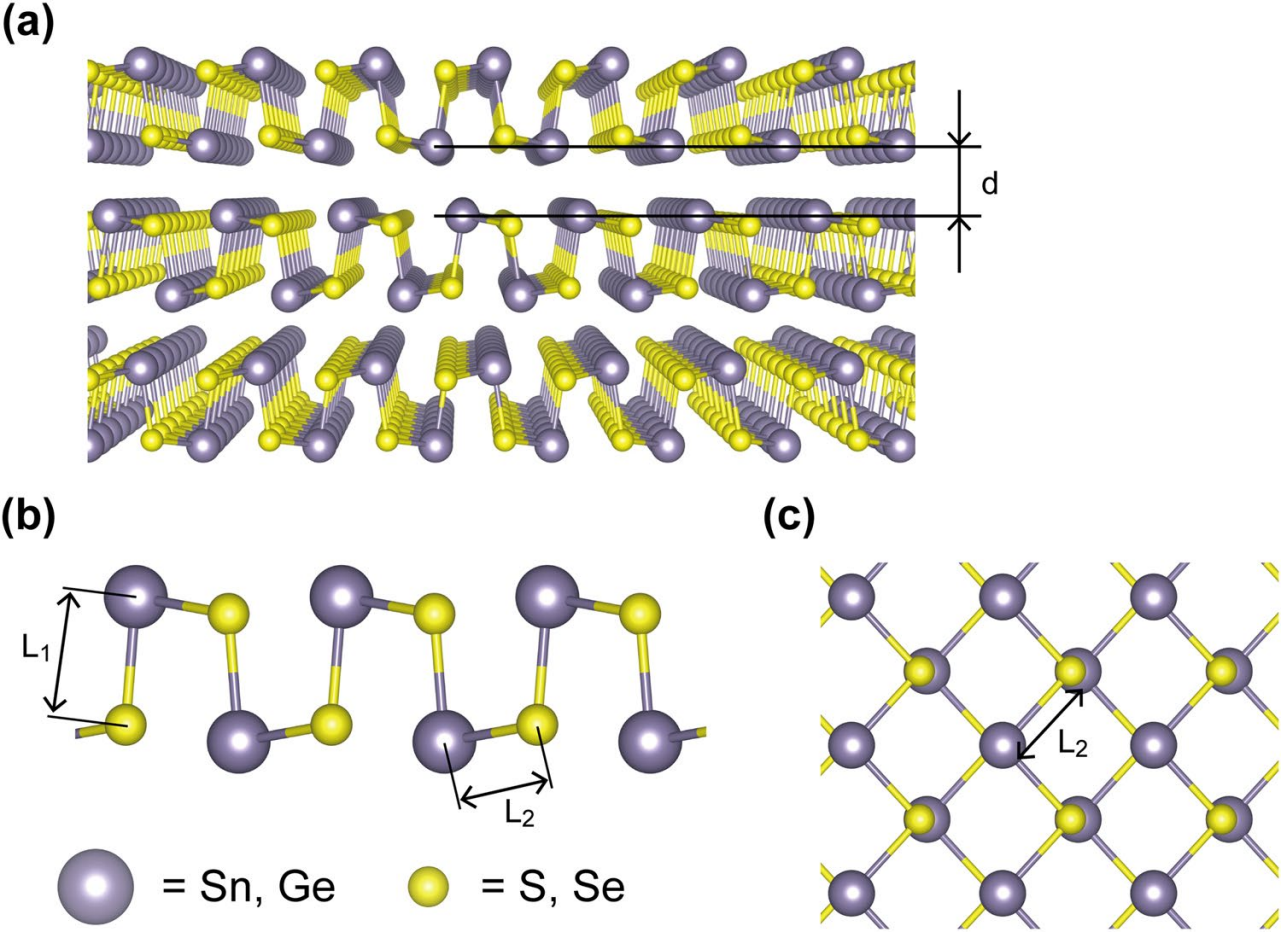
(**a**) Schematic diagram of the crystal structure of MX. It is a puckered structure like that of black phosphorus. *d* denotes the interlayer distance. (**b**) L_1_ and L_2_ represent bonding lengths of the vertical and horizontal directions of the plane, respectively. (**c**) Top view of the crystal structure.

More recently, in few-layer metal monochalcogenides (MX, M = Sn, Ge; X = S, Se, etc.), members of layered IV-VI compounds have been discovered through exfoliation from bulk structures^26–28^. The structures are the same as that of black phosphorus, which is called the puckered structure, where M and X are bonded alternatively (Fig. 1). These novel materials show intriguing electric polarization properties^26,27,29,30^. In addition, they have properties of vertical dielectric screening, resulting in changes to the electronic and optical properties^27,31,32^. On the other hand, due to their high absorption coefficient, they can be used for photovoltaic cells^3,33–38^ and show high performance. They can also be used for lithium-ion battery anodes^39,40^, because they have layered structures where Li are intercalated to space between layers, exhibiting high Li capacity. In addition, MX has attracted much attention due to the ~1 eV electronic bandgap close to bulk silicon. Thus MX has a huge potential for use in electric devices^41^. Similar to almost layer stacking structures, monolayer or few-layer MX could have a distinctive property or better performance for certain devices, like thin-film devices^42^. Recently, there have been many attempts to synthesize few-layer MX, including through the CVD method^43–48^, chemical bath deposition^38,49,50^, atomic layer deposition^18,51^, and spray pyrolysis^52^. Heterostructures with MX have also been studied as field-effect transistor device materials^48^.

However, like graphene and transition metal dichalcogenides, verifying the number of layers in few-layer MX structures is crucial when they are used as device materials. In stacking a layer, we average the length of all similar direction bonding. While MX has attracted great interest, verifying the number of layers of MX sample has not yet been sufficiently studied. For example, there has only been one case verifying the thickness of a sample using an electronic bandgap^10^; that is a good method for figuring out the thickness. However, measuring a bandgap has too many more steps than Raman and can ruin an exfoliated crystal sample. Therefore, nondestructive Raman method might be the best method, but there are no relevant reference data. In this paper, we performed Raman simulation for MX as the number of layers varied from a monolayer to bulk in order to provide standard Raman spectroscopy data. We focus on SnS, SnSe, GeS, and GeSe as representative examples for MX. Our calculations show that the Raman spectroscopy for metal monochalcogenides depends on the number of layers. We found that the red shift and blue shift occur continuously as the number of layers increases by ~100 cm^−1^. This can be explained by the fact that the shifts come from the change of the bond lengths between the metal atoms and chalcogen atoms by the change of the interlayer interaction, leading to the slight modification of a vibration mode. These results provide benchmarks for determining the number of layers of metal monochalcogenides by Raman spectroscopy analysis.

All of the calculations were carried out using density functional theory (DFT) and density functional perturbation theory (DFPT)^53^, as implemented in the PWSCF package of the QUANTUM-ESPRESSO^54^. The DFT is employed for the geometry optimization of the IV-VI layers, and the DFPT is employed for calculations of the Raman spectra, phonons, and dynamical matrices. We used norm conserved local density approximation (LDA) for pseudopotentials^55,56^, which gives consistent results with PBE-D3. The kinetic energy cutoff was taken to be 30 Ry. We use 9 × 9 × 3 and 9 × 9 × 1 k-point for the bulk and layered structures, respectively. Geometrical optimizations are performed for every single structure until the Hellman-Feynman force is below 10^−8^ Ry/Bohr. All of our calculations were converged to ~10^−4^ Ry/atom. Detail information for the optimization of the lattice vectors and the lattice constants is described in supporting information Figure S1, Figure S2, and Table S1.

We first performed calculations on the geometry optimizations for AB staked multilayered MXs (Fig. 1a) because it has been confirmed that, for MX bulks, AB stacked MX layers in bulks are the energetically most favorable configuration^57,58^. Figure 1 shows the optimized atomics structures of AB-stacked multilayer MX structures, where *L*_1_ and *L*_2_ denote the bonding lengths for out of plane and in-plane, respectively. The calculated values of the L_1_ and L_2_ and the interlayer distances for AB stacked MX multilayers are presented in Table 1, which are consistent with the values in the literature for a multi-layer^59^. For instance, L_1_ and L_2_ were calculated to be 2.56 and 2.70 Å for bilayer SnS, respectively. The bond lengths are increased as the atomic numbers of M and X increase. However, the interlayer distance, *d* is 2.64 Å (Supporting information Figure S3), slightly dependent on the type of MX layers, which corresponds to the equilibrium distance of ~3.4 Å via vdW interaction. Furthermore, the calculated values of L_1_, L_2_, and *d* as the number of layers varied from 1 to 3 are also presented in Table 1. We also carried out the calculations on the interlayer binding energy of bilayer and trilayer MXs in Table 2. For comparison, the interlayer binding energy and the interlayer distance were also calculated with other functional, i.e., meta-GGA^60^. The binding energy and the interlayer distance obtained with the LDA is slightly larger and smaller than those with the meta-GGA, respectively. We found that there are small changes in L_1_ and L_2_ as well as interlayer distance, similar to other 2D materials^12,61^.

**Table 1:** Calculated local geometry information for AB stacked MX layers as the number of layers.

Materials	# of layers	*L*_1_	*L*_2_	Δ_1_	Δ_2_	*d*
SnS	1	2.53	2.73	0	0	—
	2	2.56	2.70	0.03	−0.03	2.64
	3	2.58	2.69	0.05	−0.04	2.64
	∞	2.61	2.64	0.08	−0.09	2.57
SnSe	1	2.66	2.91	0	0	—
	2	2.69	2.84	0.03	−0.07	2.79
	3	2.70	2.79	0.04	−0.12	2.71
	∞	2.75	2.77	0.09	−0.14	2.66
GeS	1	2.36	2.46	0	0	—
	2	2.39	2.45	0.03	−0.01	2.49
	3	2.39	2.44	0.03	−0.02	2.46
	∞	2.43	2.42	0.07	−0.04	2.36
GeSe	1	2.49	2.59	0	0	—
	2	2.51	2.59	0.02	0	2.80
	3	2.52	2.56	0.03	−0.03	2.58
	∞	2.55	2.56	0.06	−0.03	2.51

Δ_1_(Δ_2_) denotes *L*_1_ (*L*_2_) for a given layer minus *L*_1_ (*L*_2_) of monolayer presented in Fig. 1(b). The unit of the values below is angstroms**. ∞** means multilayer of MX. *d* indicates the interlayer distance.

**Table 2:** Calculated binding energy (E_b_) between interlayers for few-layer MXs and interlayer distance (*d*). The exchange-correlation functional is treated using LDA and strongly constrained and appropriately normed (SCAN) meta-generalized gradient approximation (meta-GGA).

Functionals	# of layers	SnS	SnSe	GeS	GeSe
LDA	2	E_b_ = 0.50 eV	0.50 eV	0.49 eV	0.38 eV
		*d* = 2.64 Å	2.79 Å	2.49 Å	2.80 Å
	3	E_b_ = 0.46 eV	0.51 eV	0.49 eV	0.39 eV
		*d* = 2.64 Å	2.71 Å	2.46 Å	2.58 Å
Meta-GGA	2	E_b_ = 0.26 eV	0.26 eV	0.28 eV	0.28 eV
		*d* = 2.97 Å	3.15 Å	2.73 Å	3.04 Å

**Figure 2:**
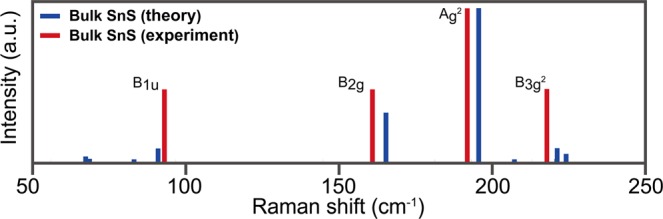
Calculated and experimental Raman spectroscopy for AB stacked multilayer SnS. The experimental data was in ref. ^62^.

We performed calculations on the Raman spectra of multilayer MX using DFPT calculation. In order to verify the computational accuracy of the DFPT, the calculated results were compared to the experimental data for AB stacked multilayer SnS^18^. We focused on the four characteristic peaks in the spectra of 53, 76, 104, and 244 cm^−1^ for monolayer SnS, because their Raman signals are remarkable and have been observed in experiments^18,62^. Remarkably, the calculated Raman spectra for SnS is strongly consistent with the experimental data (Fig. 2)^18^. Thus, we believe that our calculations on the Raman spectra for other MX layers are reliable for predicting their properties. Moreover, it was verified that there is high accuracy in Raman simulations for monolayer using the ab initio method^63^.

**Figure 3:**
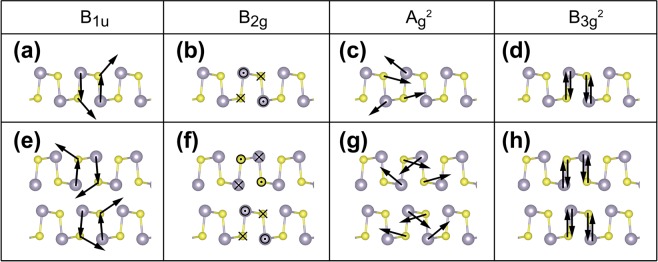
Phonon modes of monolayer ((**a**–**d**)) and multilayer (**e**–**h**) SnS: B_1u_, B_2g_, A_g_^2^, and B_3g_^2^.

We further analyzed the lattice vibrational modes of AB-stacked multilayer SnS corresponding to the characteristic Raman peaks in order to determine how the peaks are generated. We found that the phonon modes of the four B_1u_, A_g_^2^, B_2g_, and B_3g_^2^ shown in Fig. 3(e–h), respectively, are associated with the peaks for 91, 166, 196, and 221 cm^−1^ for multilayer SnS, respectively. Hereafter, we refer to the four peaks in the Raman spectra as B_1u_, A_g_^2^, B_2g_, and B_3g_^2^, respectively. Monolayer SnS has the same phonon modes in Fig. 3(a–d). For the B_1u_ phonon mode, Sn atoms move out of plane and S atoms move in the oblique plane (Fig. 3(a,e)). For the A_g_^2^ phonon mode, Sn and S atoms move in the oblique plane (Fig. 3(c,g)). For the B_2g_, and B_3g_^2^ phonon modes, the Sn and S atoms move in the same plane in the opposite direction (Fig. 3(b,d,f,h)). These modes can be approximately classified by atomic moving direction into three kinds: vertical of the MX plane, horizontal of the MX plane, and hybrid. B_2g_ is horizontal while B_3g_^2^ is vertical of the SnS plane. B_1u_ looks like a hybrid of the two. In A_g_^2^ mode, atoms have a vibration in both directions, out of plane and in-plane. However, moving in out of plane appears to be negligible. Therefore, we can classify A_g_^2^ as the horizontal mode.

**Figure 4:**
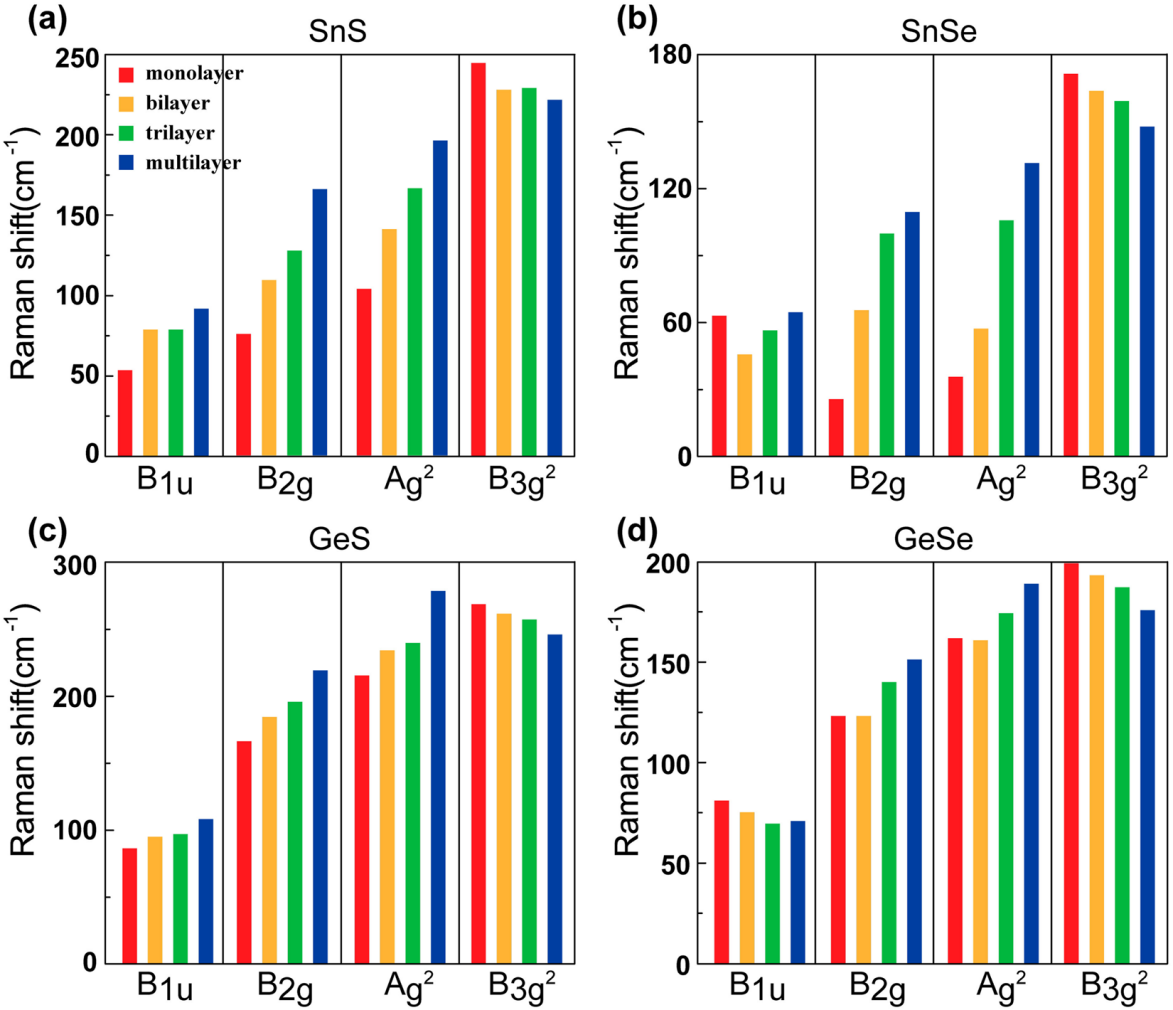
Raman shifts for four peaks in (**a**) SnS, (**b**) SnSe, (**c**) GeS, and (**d**) GeSe as the number of layers. With an increasing number of layers, Raman shifts increase or decrease depending on the type of phonon mode.

In order to investigate how the Raman spectra of few-layer MX materials depend on the number of layers, we performed calculations for few-layers MX as the number of layers varied. An attractive feature is that the Raman spectrum of SnS is dependent on the number of layers. For SnS, we found that B_1u_, B_2g_, and A_g_^2^ are a blue shift by ~40 cm^−1^, ~90 cm^−1^, and ~92 cm^−1^, respectively, while B_3g_^2^ are red shift by ~23 cm^−1^.(Fig. 4(a)) Since Raman peaks are naturally split by stacking layers, we selected the most significant peak among several split peaks. If certain peaks are degenerate in the same position, the measured intensity is higher than that of the theoretical prediction. Increasing the number of layers yields a red shift for B_3g_^2^ and a blue shift for B_2g_ and A_g_^2^. These trends appear in all four MX except for B_1u_. For the SnS and GeS layers, the B_1u_ mode shows a blue shift. For GeSe, the B_1u_ mode has a red shift trend, while it does not show any shift trend for SnSe. For other peaks, the changes appear to be sufficient to distinguish the number of layers of samples. On the other hand, the interaction addition as the number of layers is significant in a vertical direction compared with a horizontal direction because The thickness with respect to the vertical to the plane of MXs is increased as the number of their layers increases because L_1_ increases as the number of layers increases (see Table 1). Therefore, the frequency shift in a complete vertical mode, i.e., B_3g_^2^ is more significant than that other modes as shown in Fig. 4.

**Figure 5:**
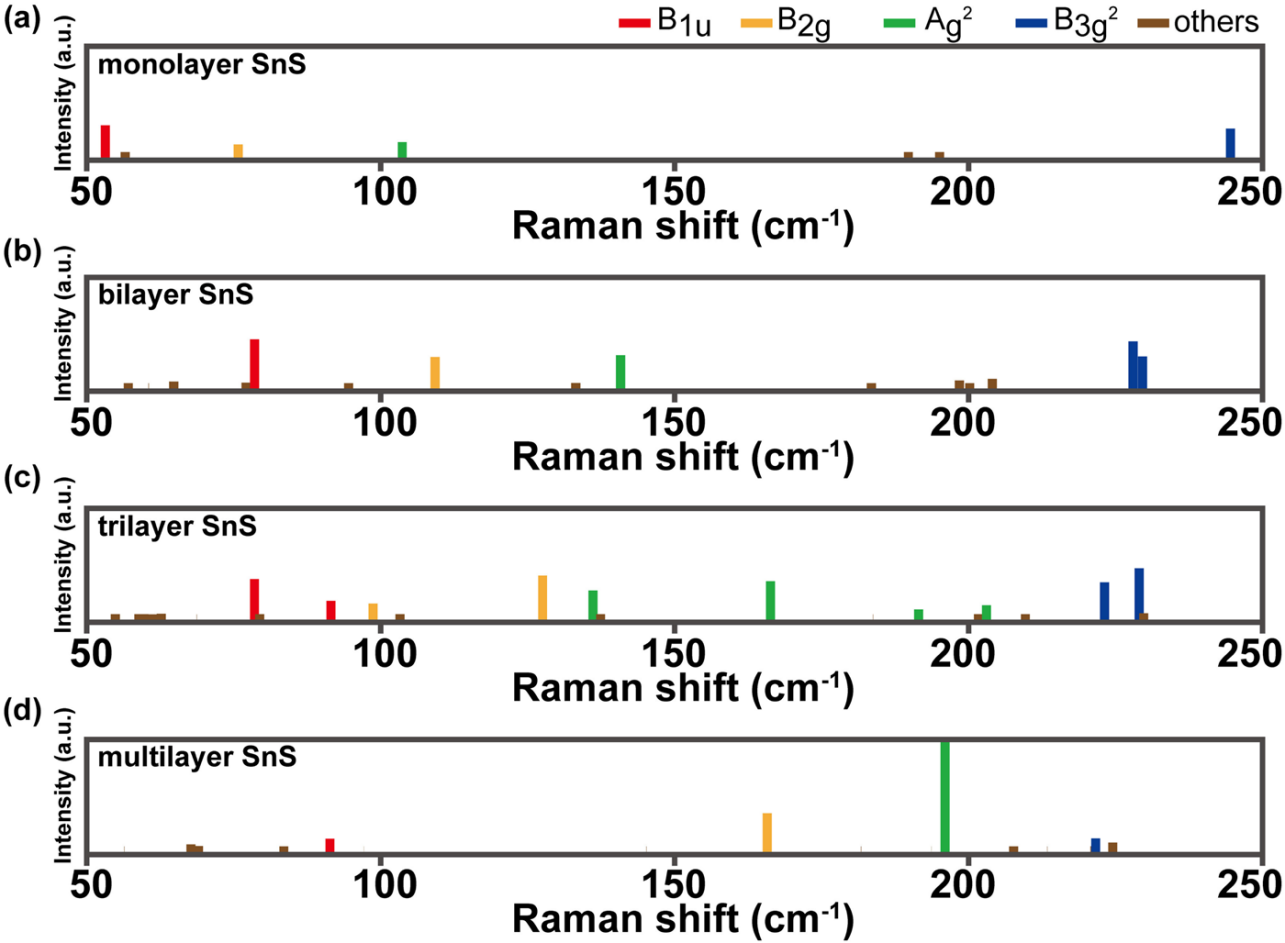
Calculated Raman spectroscopy of AB stacked few-layered SnS as the number of layers: (**a**) monolayer, (**b**) bilayer, (**c**) trilayer, and (**d**) multilayer SnS.

Moreover, we observe that the tendency depends on the direction of phonon mode, horizontal or vertical. In Fig. 5, the horizontal modes (such as B_2g_), in-plane modes, appear as the blue shift tendency, while the vertical modes (such as B_3g_^2^), out of plane modes, appear as the red shift trend when increasing the number of layers for SnS. This relation is consistent across the four kinds of MX.

We investigate what causes the difference between red and blue shift trends. In order to determine the blue and red shifts of the Raman spectroscopy, we investigated the local geometry of them with varying numbers of layers. The calculated values of the bond lengths (L_1_, L_2_) between L_1_, L_2_, and interlayered distance as the number of layers varies are presented in Table 1. Importantly, we found that there is a small change of length for unit cell vectors, as in other 2D materials^12,61^. Further, there are certain changes of atomic bonding length from interlayer interactions. This can change the lattice vibration motion, which might be one of the causes of the shift tendency. We can understand that the change of bond lengths can be interpreted by the change of the spring constant using a simple harmonic approximation. Hence, when a bonding length is decreasing, a frequency is increased. This is the reason for why Raman shift frequency, a peak position, is changed according to the number of layers. According to the number of layers, the changes for L_1_ and L_2_ are shown in Table 1. With increasing the number of layers, L_1_, an average bond length of the vertical direction, is increasing, while L_2_, an average bond length of the horizontal direction, is decreasing. In other words, with increasing the number of layers, the vertical mode has a red shift and the horizontal mode has a blue shift. This relation holds for all our studied MXs.

**Figure 6:**
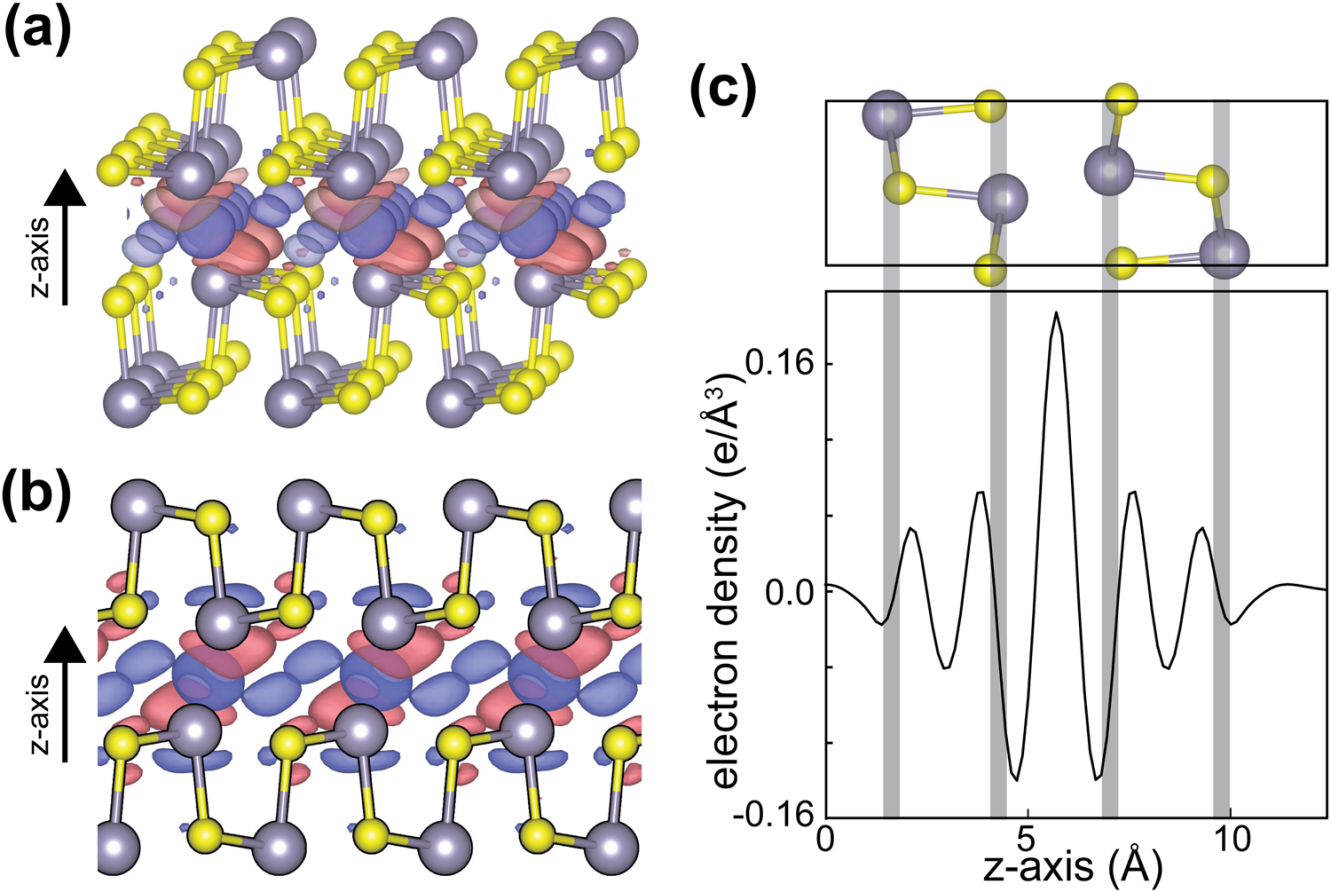
Calculated charge density difference between interlayers: (**a**) Oblique view (**b**) Side view. The red and blue colors indicate the accumulation and depletion of electrons, respectively. (**c**) In-plane projected charge density along with z direction.

In order to understand the origin of the bond lengths of few-layer MXs resulting in the shift of the phonon energy, we investigate the bond between layers. We found that this change of the lattice parameter via the interaction between layers stems from the weakly covalent bonding between interlayers, as shown in Fig. 6, where the charges transfer between interlayers. This differs from the bond between interlayer of few-layer vdW materials like graphene or MoS_2_. In addition, the binding energy of the layers is calculated to range from 0.2 eV to 0.5 eV, slightly dependent on the type of sheets (Table 2), which is greater than the value of a few ten meV in graphite and MoS_2_^64–66^. This result is consistent with those of previous studies^67,68^. Therefore, we believe that, because of the stronger interaction between layers than other vdW materials, the Raman shifts as the number of layers occur.

The relation between Raman shifts (Δ*v* (cm^−1^)) and the number of layers for MXs is shown in Fig. 4. We confirmed that this relationship remains very consistent for A_g_^2^, B_3g_^2^, and B_2g_. The data could almost be fitted linearly by a reciprocal number of layers as follows:

1 $$\Delta {\nu }_{i}\,({{\rm{cm}}}^{-1})=\frac{{a}_{i}}{N}+{b}_{i},$$

where the subscript *i* indicates the index of phonon modes, *a*_*i*_ and *b*_*i*_ are fitting coefficients for a given *i* and *N* indicates the number of MX layers. The calculated coefficients for MX layers were calculated by a linear fitting (Supporting information Figure S1), which are presented in Table 3. The negative and positive values of *a*_*i*_ mean red and blue shifts as *N* increases, respectively. This will be very beneficial in experiments to know the number of layers for a sample. Even though some shifts are of a small value per layer, like GeSe, modern Raman equipment has sufficient resolution (<1 cm^−1^) in room temperature to identify them^69^. In addition, for all examples, there are more than two rapid shift peaks, enough to identify thickness. The blue shift peaks, the horizontal modes, might be useful indicators for verifying the number of layers for almost MX. Another important point is that we propose a method of distinguishing the number of layers by comparing predicted Raman shift using Eq. (1) with experimental results of the Raman spectrum.

**Table 3:** Coefficients (*a*_*i*_*, b*_*i*_) to Eq. (1) for each phonon mode. The unit of the values below is cm^−1^.

	B_1u_	B_2g_	A_g_^2^	B_3g_^2^
**SnS**	(−37.7, 92.5)	(−88.9, 160.2)	(−93.3, 194.3)	(22.6, 220.3)
**SnSe**	(−1.2, 58.0)	(−88.3, 115.6)	(−99.4, 128.0)	(23.4, 149.6)
**GeS**	(−21.4, 106.8)	(−52.2, 215.6)	(−59.9, 269.8)	(21.9, 248.3)
**GeSe**	(11.2, 69.1)	(−28.8, 148.0)	(−26.8, 184.3)	(23.0, 178.4)

In conclusion, we have done the first-principles calculations for Raman spectroscopy based on the density functional theory. We found important things for a shift of the Raman peaks of metal monochalcogenide (MX), as its the number of layers. (1) Raman spectroscopy is considerably dependent upon the number of layers. (2) We have discovered the shifting or red trend of Raman peaks as the number of layers due to the change in atomic bonding length along with the direction of phonon. (3) We believe that our graphs for Raman peaks are useful benchmarks for identifying the number of layers of an arbitrary MX sample. Our results propose a method for distinguishing the number of layers in recently-reported few-layer MXs.

## Supplementary information

Supplement Information1
