# Supplementary material for: Raman Spectra Shift of Few-Layer IV-VI 2D Materials
Source: Sci Rep. 2019 Dec 20;9:19826. doi: 10.1038/s41598-019-55577-x (PMC6925276; doi:10.1038/s41598-019-55577-x)
Supplement: Supplementary file 1 — Supplement Information1 [file 41598_2019_55577_MOESM1_ESM.pdf]

Supporting Information for

Can Raman Spectra Shift Distinguish the Layer Number of Few-Layer IV-VI 2D Materials?

Minwoo Park[1, 2], Jin Sik Choi[2], Li Yang [1,], and Hoonkyung Lee [2,*]*

[1]*Department of Physics, Washington University in St. Louis, St. Louis, Missouri 63136, USA*

[2]*Department of Physics, Konkuk University, Seoul 05029, Korea*

*To whom correspondence should be addressed: Li Yang (lyang@physics.wustl.edu), Hoonkyung Lee (hkiee3@konkuk.ac.kr)

Section S1. Linear fitting for Raman peaks as reciprocal number of layers:

We linearly fitted the Raman shift of the four Raman modes for few-layer SnSe, SnS, GeSe, and GeS obtained through the DFT calculations to Eq. (1) as shown in Figure S1. The coefficients, *a* and *b*, of the shift for the layers are presented in Table 3.

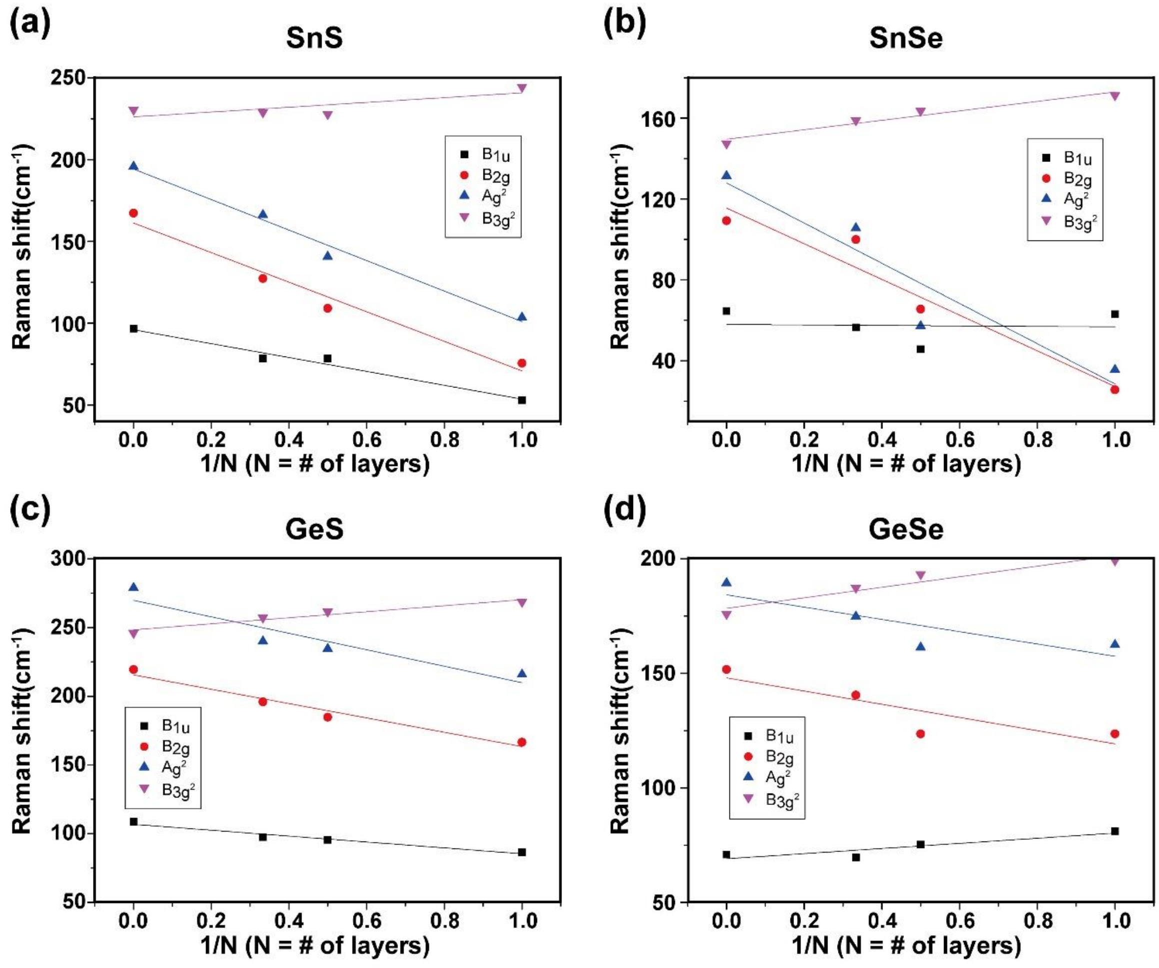

Figure S1. Linear fitting graph for the Raman shifts as the reciprocal number of layers with Eq. (1). The coefficients for fitting are presented in Table 3.

Section S2. The lattice vectors of few-layer MX layers:

We defined the lattice vectors of from few-layer MXs as follows:

$$\vec{A}_1 = a\hat{x}$$
$$\vec{A}_2 = b\hat{y} \quad , \qquad \text{R(1)}$$

where *a* and *b* indicate the lattice constants for finite layer MXs. We carried out calculations on the total energy of the systems as a function of the lattice constants, i.e., *a* and *b*. as shown in Figure S1. The lattice vectors were optimized with the energy minimization as the lattice constants of all the-VI materials vary with the accuracy of the energy minimization smaller than 10^{-2} eV per cell. For instance, we performed calculations on the total energy map of SnS as a function of the lattice constants, *a* and *b* (Figure S2(a)). *a* and *b* were determined by a minimum point of the energy contour (Figure S2)). The calculated lattice constants for the all MX layers are presented in Table S1.

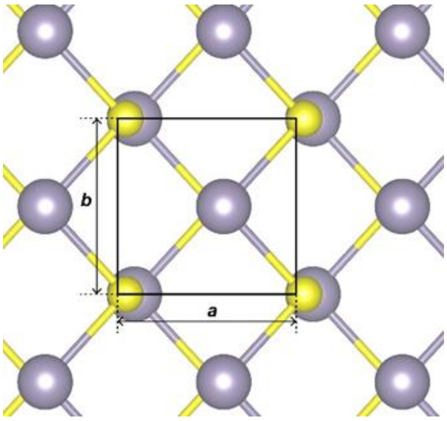

Figure S1. The lattice vectors of few-layer MXs, where *a* and *b* indicate the lattice constants of the MX layers defined by Eq. R(1).

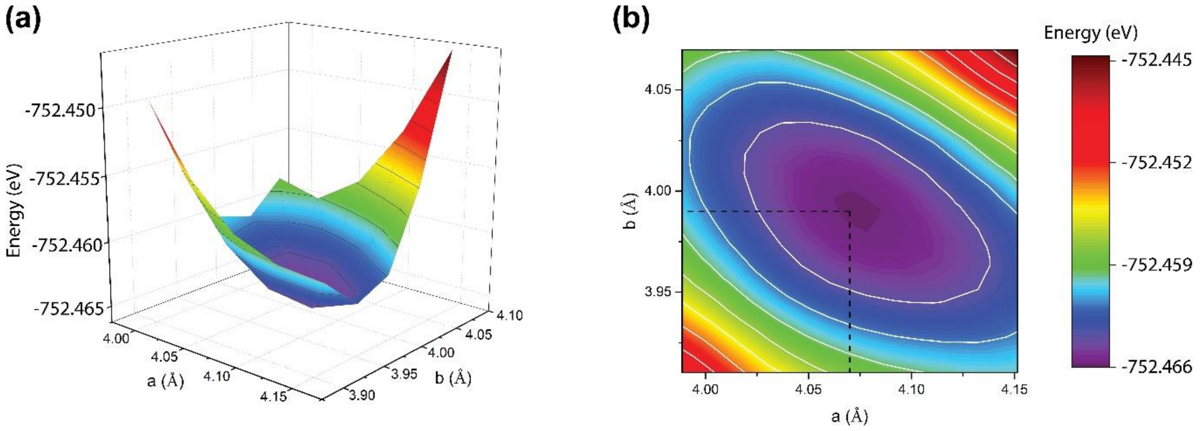

Figure S2. (a) 3D energy color map as a function of the lattice constants of monolayer SnS, a and b, defined by the Eq. R(1). (b) 2D energy color map with the contour line. The contour interval is 0.002 eV and the two graphs share the same color bar.

Table S1. The calculated lattice constants of MX layers.

# of layer	SnS	SnSe	GeS	GeSe
**1**	*a*=4.07 Å, *b*=3.99 Å	*a*=4.22 Å, *b*=4.20 Å	*a*=4.03 Å, *b*=3.66 Å	*a*=4.05 Å, *b*=3.90 Å
**2**	*a*=4.07 Å, *b*=3.99 Å	*a*=4.22 Å, *b*=4.20 Å	*a*=4.03 Å, *b*=3.66 Å	*a*=4.18 Å, *b*=3.86 Å
**3**	*a*=4.01 Å, *b*=3.93 Å	*a*=4.32 Å, *b*=4.11 Å	*a*=4.03 Å, *b*=3.66 Å	*a*=4.18 Å, *b*=3.86 Å

Section S3. The interlace distance of few-layer MX layers

To obtain the energy of few-layer MXs as the interlayer distance (*d*), we performed the full relaxation calculations on MX layers with the optimized lattice constants of the materials as the interlayer distance varies. For example, there is a minimum energy for bilayer SnS at *d*=2.64 Å as shown in following Figure 3S, which corresponds to the equilibrium interlayer distance. Using this method, the interlayer distance in our systems is calculated to be ~2.5–2.8 Å. This is smaller than ~3.2–3.4 Å for the van der Waals interaction, i.e., TMDs because, unlike van der Waals materials, a weakly covalent bonding contributes to the interlayer interaction between layers (Figure 6).

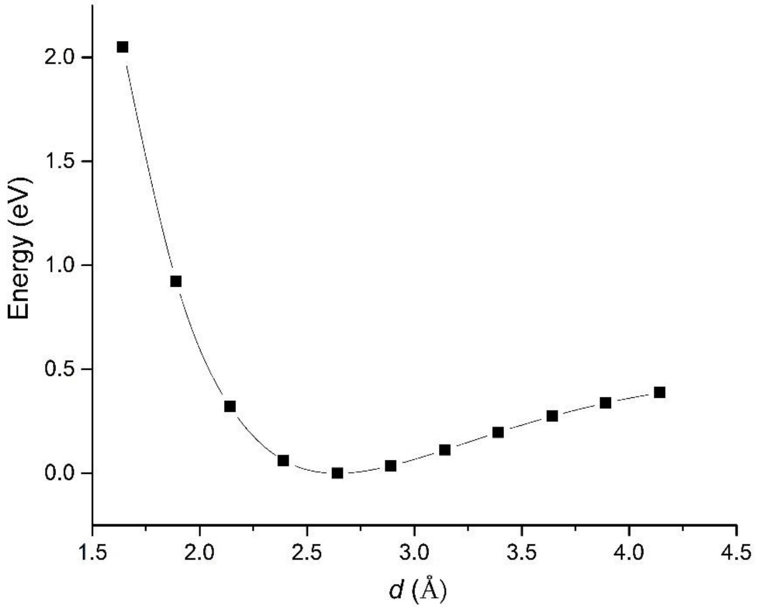

Figure S3. Energy curve by interlayer distance(d) for bilayer SnS. There is a minimum energy value at *d*=2.64 Å.
